# Mycotic Aneurysm of the Thoracoabdominal Aorta: A Diagnostic Challenge

**DOI:** 10.7759/cureus.40894

**Published:** 2023-06-24

**Authors:** Maham Shahid, Benjamin Phan, Vincent Tir, Corey Engel, Jessica El-Bahri

**Affiliations:** 1 Internal Medicine, HCA Florida Orange Park Hospital, Orange Park, USA; 2 Radiology, University of Florida College of Medicine, Jacksonville, USA

**Keywords:** ruptured aneurysm, illicit fentanyl, aorta-aortic by pass, diagnostic delay, renal abscess, methicillin resistant staphylococcus aureus (mrsa), intravenous drug use (ivdu), mycotic aneurysm of thorax-abdominal aorta

## Abstract

Mycotic aortic aneurysms (MAAs) are a rare form of aortic aneurysms that are associated with catastrophic outcomes if not diagnosed and treated on time. However, MAAs are a diagnostic challenge owing to their often nonspecific presentation. In this study, we present a case of a 42-year-old female with a pertinent history of intravenous drug use who presented with generalized body pain for two weeks and was found to have a mycotic thoracoabdominal aortic aneurysm (TAAA) extensively involving adjacent structures, including lungs with pleural cavity and upper renal pole. Not only does this case highlight the difficulty in early diagnosis and complex pathology of a mycotic TAAA, but it also illustrates the multidisciplinary approach required to effectively treat them.

## Introduction

Mycotic aneurysms were first described by William Osler in 1885 when he noted a patient with endocarditis who developed multiple aortic aneurysms with the appearance of *fresh fungus vegetations* and called it mycotic endarteritis [1]. In present times, the term *mycotic* is used to describe an arterial aneurysm that develops once an infected arterial wall becomes dilated. These infective aneurysms may develop in any vessel, including the aorta, femoral, and intracranial or visceral arteries. Less than 3% of all aortic aneurysms are mycotic; they most commonly develop in the abdominal aorta and occur least commonly in the thoracic aorta [2]. The latter, of which, confers a worse prognosis [3]. Most reported series have fewer than 50 patients with mycotic aneurysms of the thoracic aorta [4]. The pathogenesis of infected arterial aneurysms is most frequently caused by bacteremic seeding, although it may also occur from direct inoculation, contiguous infection, and septic emboli in a patient with endocarditis [5]. While mycotic aortic aneurysms (MAAs) have been discussed in the literature, diagnosing MAA remains challenging due to its nonspecific clinical presentation. Here, we present a unique case of a mycotic thoracoabdominal aortic aneurysm (TAAA) with extensive involvement of adjacent viscera. 

## Case presentation

A 42-year-old female with a past medical history of intravenous (IV) opioid use disorder and hypertension presented to the hospital with a two-week history of generalized body pain. Her symptoms were associated with excruciating left hip pain and a cutaneous abscess on her left gluteal region and right cubital fossa. She reported these abscesses had been growing for two weeks and started draining spontaneously three days before admission. She has had multiple cutaneous abscesses in the past, most commonly on her upper extremities in areas with evidence of track marks. She reported that she last injected fentanyl three weeks before her presentation.

On admission, the patient was afebrile and her blood pressure was elevated at 224/98 mmHg. Laboratory values were significant for leukocytosis, thrombocytosis, and elevated inflammatory markers, as shown in Table 1. The urine drug screen was positive for oxycodone and benzodiazepines. Urinalysis and electrocardiogram were unremarkable. Chest radiography revealed patchy left basilar airspace opacities with concern for developing pneumonia. She was initiated on sepsis protocol with IV fluids and broad-spectrum IV antibiotics - vancomycin and cefepime. Bedside incision and drainage were performed on the gluteal abscess, which drained 280 mL of purulent fluid. Wound cultures were sent, which subsequently grew methicillin-resistant *Staphylococcus aureus* (MRSA) on hospital day 3. A transthoracic echocardiogram demonstrated normal systolic function without valvular abnormalities. 

**Table 1:** Laboratory Investigations. CRP, C-reactive protein; WBC, white blood cells

Laboratory tests	Patient values	Reference values
WBC	23.9 × 10^3^ microL^–^^1^	4.0 × 10^3 ^to 10.5 × 10^3^ microL^–^^1^
Hemoglobin	11 g/dL	11.2-15.7 g/dL
Platelets	686 × 10^3^ microL^–^^1^	150 × 10^3^ to 400 × 10^3^ microL^–^^1^
Creatine kinase	29 U/L	34-145 U/L
High-sensitivity Troponin I	10.24 pg/mL	3-45.20 pg/mL
Lactic acid	3.7 mmol/L	0.4-2.0 mmol/L
CRP	170 mg/L	<10 mg/L

**Figure 1:**
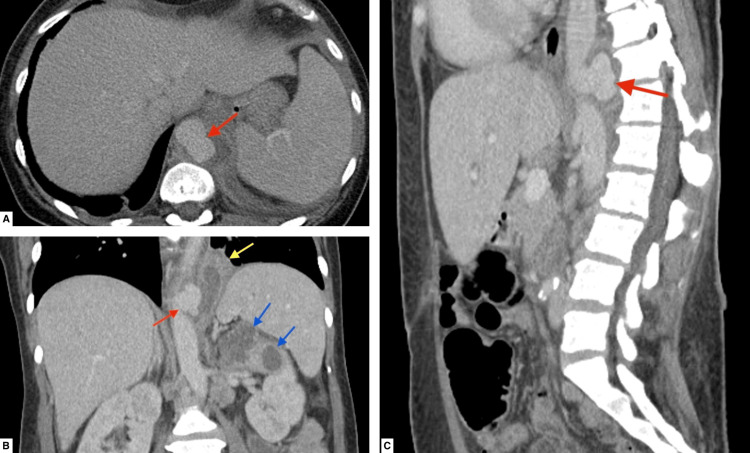
CT scans of abdomen and pelvis with IV contrast showing saccular aneurysm of thoracoabdominal aorta (red arrows in A-C), complex fluid in the left pleural space tracking into the major fissure (yellow arrow in B), and left upper renal pole abscesses (blue arrows in B). IV, intravenous; CT, computed tomography

Despite initial negative blood and urine cultures, administration of broad-spectrum antibiotics, and re-evaluation of the abscess site by general surgery, the patient continued to have a high-grade fever, leukocytosis, and elevated C-reactive protein (CRP). On hospital day 4, the patient developed unbearable left-sided flank pain with costovertebral angle tenderness. A noncontrast computed tomography (CT) scan of her abdomen and pelvis demonstrated pyelonephritis with suspicion of a renal abscess. Given the abnormal renal findings, a CT abdomen and pelvis with IV contrast were obtained, which revealed a saccular pseudoaneurysm of the distal thoracic aorta with adjacent inflammatory changes, fluid with abscess of the medial aspect of the left pleural space, complex fluid tracking into the major fissure, and multiple left upper pole renal abscesses (Figures 1A-1C).

Cardiothoracic surgery evaluated the patient and requested CT imaging of the chest with IV contrast; it showed findings consistent with previous imaging as well as bilateral basilar pulmonary nodules and bilateral axillary lymphadenopathy. The repeat blood culture drawn on hospital day 4 grew MRSA, and a diagnosis of mycotic TAAA was made. Given these findings and the advanced interventions that were required, the patient was transferred to a tertiary care facility for further management. At the facility, repeat imaging revealed a contained rupture of the mycotic TAAA. A transesophageal echocardiogram was negative for vegetation. She underwent two-staged surgical interventions, which included visceral debranching with ileo-hepatic, superior mesenteric artery, and renal artery bypass. Three days later, she had a successful repair of the mycotic TAAA with an aorto-aortic bypass.

## Discussion

The true prevalence of MAAs is unknown but accounts for only 0.7% to 2.6% of all aortic aneurysms [6,7]. Furthermore, TAAAs account for 5% to 10% of aneurysms of the aorta, and mycotic aneurysms of the thoracoabdominal aorta represent 1.8% of all TAAAs [8]. After an extensive literature review, very few cases of mycotic TAAAs with contiguous involvement of viscera have been reported. Specifically, in this patient, she had a saccular aneurysm of her thoracoabdominal aorta, complex fluid in her pleural space tracking into the major fissure of the left lung, and multiple left renal abscesses. Unlike the patient of this study, up to 60% of all mycotic aortic aneurysms may present as ruptured [7].

MAAs, simply put, are a conundrum. They present with a multitude of vague findings, including but not limited to fever, abdominal or back pain, shock, and nonspecific laboratory results (leukocytosis, elevated inflammatory markers, etc.) [9]. In our case, the patient presented with generalized body pain, multiple abscess sites, and active IV drug use disorder. Some confounding factors may have clouded the urgency of our diagnostic workup as there was a concern for active illicit drug withdrawals contributing to fluctuating hemodynamics and generalized body pains. Her septic picture was likely secondary to her initial presentation with large subcutaneous abscesses and suspicion of pneumonia as per the chest radiograph. Yet, even after receiving several days of IV antibiotics, she remained persistently septic, which was attributed to subtherapeutic troughs and inadequate medication dosing.

The responsible microorganism is identified in almost 80% of the cases with gram-positive cocci (*S. aureus* and *Streptococcus *spp.) accounting for 28% to 71% of cases and gram-negative rods (most commonly *Salmonella *spp.) responsible for up to 50% of all cases [4,9]. Although most MAAs have positive blood cultures, negative blood cultures are insufficient to rule out MAAs as they can be negative in 25% to 50% of the patients [2,10].

Detection of MAAs requires multidetector CT, multislice CT angiography with three-dimensional (3D) reconstruction, and magnetic resonance imaging (MRI). Some alternative noninvasive modalities that are gaining favor include MRI with gadolinium enhancement and nuclear medicine studies, fluorodeoxyglucose positron emission tomography, as well as nuclear gallium scanning [10]. These imaging modalities not only help diagnose but also characterize the MAAs and obtain vascular mapping to formulate a treatment plan in confirmed cases [11]; in our patient’s instance, these radiographic studies helped the medical care team plan a two-staged surgical intervention.

Management comprises a multidisciplinary approach of intensive antibiotic therapy frequently combined with surgical or endovascular interventions [4]. Although there is no data to support the specific duration of antibiotic therapy, the consensus agrees to at least six weeks of antibiotic therapy [10]. A study by Bernal et al. suggested life-long antibiotic therapy in patients who underwent endovascular repair with positive cultures obtained from tissue samples [12]. The decision for lifelong antibiotics can be challenging, especially in cases where IV drug use disorder is involved, and such patients would require drug rehabilitation programs. In our case, the patient remained hospitalized for continued IV antibiotics for six weeks.

If left untreated, MAAs are almost invariably catastrophic from hemorrhage secondary to rupture [13,14]. According to a systematic review of 28 studies, endovascular repair appears to be associated with superior short-term survival without late disadvantages, compared with open surgical repair, which is associated with a mortality rate of up to 40% [15,16].

## Conclusions

Mycotic TAAAs are uncommon but universally fatal. This case highlights an uncommon presentation of an MAA with extensive involvement of the surrounding structures and serves to illustrate the difficulty in early diagnosis. In this patient, nonspecific complaints, initial negative blood cultures, concern for opiate withdrawal, and pain-seeking behavior distracted from early recognition of a mycotic TAAA. Despite the availability of state-of-the-art imaging modalities, the diagnosis of MAA remains clinically challenging, and a high index of suspicion is required due to its ambiguous presentation.
